# Evaluation of point-of-care hemoglobin testing vs. central laboratory measurements in neonates: a prospective study in neonatal intensive care unit

**DOI:** 10.3389/fped.2026.1735203

**Published:** 2026-02-12

**Authors:** Vivian Chang, Wei Hou, Echezona T. Maduekwe

**Affiliations:** 1 Department of Pediatrics, Neonatology, Stony Brook Children’s Hospital, Stony Brook, NY, United States; 2 Family, Population and Preventive Medicine, Stony Brook University Hospital, Stony Brook, NY, United States

**Keywords:** blood gas analysis, hemoglobin concentration, neonatal intensive care, neonates, point-of-care testing

## Abstract

**Introduction:**

Point-of-care (POC) testing for blood gas analysis is commonly used for neonates in the Neonatal Intensive Care Unit (NICU) to determine blood gases using small blood volumes. This helps reduce blood loss and speeds up treatment. However, many clinicians remain skeptical about the accuracy of POC hemoglobin results. Our study evaluated the agreement between POC testing and laboratory measurements of hemoglobin concentration in infants born at >28 weeks gestational age.

**Methods:**

This prospective cohort study analyzed 187 paired blood samples from infants born after 28 weeks of gestation admitted to the NICU between July 2023 and December 2024. Hemoglobin levels from paired blood samples were measured using two different Methods: a POC gas analyzer and a laboratory analyzer. The laboratory analyzer (Sysmex XN-9100®) requires a minimum of 500 µL of blood, while the POC analyzer (ABL90 Flex Radiometer®) only needs 65 µL. A Bland-Altman plot was utilized for statistical analysis to assess the agreement between the two methods and to identify any systematic differences.

**Results:**

Of 197 eligible patients, 187 were enrolled in the study, with a mean gestational age of 36.6 weeks (± 3.2) at birth. There was no significant difference between the results from laboratory arterial-venous samples (16.14 g/dL) and POC testing (16.04 g/dL; *p* = 0.34). However, capillary samples from the POC testing showed significantly higher results (*p* = 0.01) than the laboratory results. The Bland-Altman plot revealed a mean difference of 0.09 for arterial-venous samples and −0.41 for capillary samples when comparing POC results with laboratory results.

**Conclusion:**

Point-of-care testing for hemoglobin analysis using arterial-venous samples may be an alternative method for quantifying hemoglobin in neonates born at a gestational age >28 weeks. However, due to notable discrepancies between capillary POC results and laboratory results, it is essential to exercise caution when interpreting hemoglobin measurements obtained from capillary POC testing.

## Introduction

Frequent blood loss due to phlebotomy is a significant contributor to anemia in critically ill infants (1). This condition poses serious risks, particularly concerning oxygen delivery to vital organs. A decrease in oxygen delivery increases the risk of severe complications, such as necrotizing enterocolitis and retinopathy of prematurity (2, 3). As a result, hemoglobin levels are routinely monitored when infants are admitted to the NICU.

Nevertheless, there is a paradox in the repeated monitoring of hemoglobin levels: the process of blood sampling can itself lead to further anemia (4, 5). In our NICU, we limit blood draws for laboratory hemoglobin evaluations to 0.5 mL per draw for each neonate, regardless of gestational age or weight. Unfortunately, this limit is often exceeded, particularly when blood samples become clotted, necessitating additional evaluations. A study conducted by Widness et al. in 2005 highlighted this concern, estimating an iatrogenic blood loss of 70 mL/kg over two weeks in 47 preterm infants (6). This finding underscores the delicate balance between necessary monitoring and potential harm.

Laboratory hematology analyzers are considered the gold standard for evaluating hemoglobin levels. However, the need for larger blood volumes and frequent sampling in neonates increases the risk of iatrogenic anemia (1). Rapid access to hemoglobin results is crucial for informed clinical decision-making, especially for neonates who may deteriorate due to undetected blood loss. Delays in obtaining laboratory results can significantly impact clinical decisions, underscoring the need for more efficient testing methods. While the normal hematologic values for neonates, whether capillary or venous, have not been distinctly defined, it is well established that capillary blood tends to have higher hemoglobin values than venous blood (7). Previous studies have compared hemoglobin levels from different blood sources (e.g., capillary vs. venous) and have suggested that POC analyzers—devices that perform tests outside traditional laboratory environments with smaller blood volumes—provide a promising alternative to standard laboratory hemoglobin testing (8–10). However, there is limited information on whether hemoglobin levels differ between the same or similar blood sources when using different testing techniques.

Reducing the volume of blood required for hemoglobin testing while maintaining clinical accuracy can help minimize the negative consequences of blood loss in infants. This is particularly important for neonates born at a gestational age of more than 28 weeks without an *in-situ* umbilical arterial or radial artery catheter, as capillary blood draws are easily accessible and minimally invasive (11). However, despite the widespread availability of on-site blood gas analyzers in neonatal units, reliable information on the accuracy of hemoglobin values obtained from these devices remains lacking. While it is known that capillary blood generally exhibits higher hemoglobin levels than venous blood in this population (12), to our knowledge, no study has prospectively compared hemoglobin values obtained from laboratory testing and POC testing using the same or similar sampling source in neonates. Therefore, we aim to evaluate the agreement between POC testing and laboratory measurements of hemoglobin concentration from the same or a similar sampling source in neonates born at gestational ages >28 weeks. We hypothesize that hemoglobin values obtained from POC testing will be identical to those obtained from laboratory analyzers.

## Materials and methods

This prospective observational study, conducted at Stony Brook Children's Hospital in New York from July 2023 to December 2024 with waivers of consent, was approved by the Institutional Review Board. The study involved 187 paired blood samples collected from infants born after 28 weeks of gestation admitted to the NICU. All infants presented with respiratory distress and, according to standard care procedures, underwent a complete blood count and gas analysis upon admission.

Blood samples for POC testing and laboratory measurements were collected simultaneously from the same source or a similar one within an hour of life. A neonatal lancet (1 mm × 2.5 mm) was used for heel pricks to obtain POC and laboratory capillary specimens. Alternatively, a 25G blood collection set (0.5 mm × 19 mm × 305 mm) was used for arterial and venous specimens. Capillary blood specimens for POC testing were collected in heparinized plastic capillary tubes, while arterial and venous POC specimens were collected in a heparinized 1 mL blood gas syringe. Laboratory hemoglobin specimens were collected in a 1 mL tube containing ethylenediaminetetraacetic acid (EDTA) and were gently inverted several times before transport to the laboratory. Capillary samples in the capillary tubes were mixed using a magnet and a metal mixing bar. The purpose of the inversion and the mixing of blood samples by the nurses or the respiratory therapists was to ensure uniformity of the specimen. The specimen for POC hemoglobin testing requires approximately 2 min to arrive at the POC evaluation area in the NICU, while the laboratory hemoglobin specimen is transported via the TransLogic® pneumatic tube system (Swisslog Healthcare, Broomfield, CO, USA), taking around 95 s to reach the specimen collection center and an additional 15 min to arrive at the hematology laboratory.

We defined hemoglobin turnaround time as the interval, in minutes, from sample collection to test result availability. Sampling times are documented in the electronic medical record by the nurse or nurse practitioner performing the procedure. Result times are recorded by the respiratory therapist for POC hemoglobin or by the laboratory technician for central hemoglobin.

Previous studies, including those by Anezi et al. (13), have shown a strong positive correlation between POC testing of arterial hemoglobin levels and laboratory venous hemoglobin levels. Therefore, samples obtained from arterial and venous sources were categorized as “arterio-venous.” Paired results were compared if collected at the same time or within one hour of each other and were of the same type: arterial with arterial, venous with venous, and capillary with capillary. Only a pair of results per infant was included in the analysis to eliminate bias from individual differences.

For laboratory hemoglobin analysis, we utilized the Sysmex XN-9100 (Sysmex Corporation, Kobe, Japan), which measures hemoglobin concentration by photometry at a wavelength of 555 nm using the cyan-methemoglobin method. This method requires a minimum of 500 microliters of blood for arterial, venous, and capillary samples. Additionally, 65 microliters from each blood sample were analyzed using the ABL90 FLEX Plus POC instrument (Radiometer Medical ApS, Brønshøj, Denmark), which employs spectrophotometry to measure hemoglobin levels and was operated by trained respiratory therapists. The laboratory analyzer was operated in accordance with the manufacturer's instructions and maintained through routine quality control measures. These included automated daily control material testing, ongoing patient data monitoring, and specialized automated maintenance routines to ensure accuracy. The ABL90 FLEX Plus features Automatic Quality Management for continuous quality control and checks. Of note, at our institution, the coefficient of variation for the Sysmex XN-9100 is equivalent to that of the ABL 90 Flex Plus (both <4.0%).

Infants were excluded from participation if they had known hemoglobinopathies, were undergoing hypothermia therapy for hypoxic-ischemic encephalopathy, had samples collected from different sources or similar sources more than one hour apart.

All enrolled patients were categorized into two groups based on the source of sample collection: arterio-venous samples and capillary samples. This structured approach reflects our clinical classifications of blood samples.

## Study outcomes

The primary outcome measure was the difference between hemoglobin levels obtained via POC testing and those from the central laboratory in infants born at gestational ages >28 weeks. We also assessed the time required to obtain these results.

### Statistical analysis

We estimated that a total of 187 infants would be needed for the study to detect a mean difference of 2 g/dL between POC testing and laboratory measurement of hemoglobin levels (14). This estimation was based on a 95% confidence level and 80% statistical power. To assess analytical accuracy, we used Student's paired t-test to compare paired samples from the same patient, and we verified the normality of the measurements by assessing the distribution of differences between paired observations with Q-Q plots. Additionally, we estimated bias and 95% confidence intervals (calculated as bias ± 1.96 × standard deviation of bias) using Bland-Altman analysis, which involved comparing the differences in paired hemoglobin values on the vertical axis against the averages of the paired hemoglobin values on the horizontal axis. We fixed an acceptable limit of agreement at 1 g/dL according to the existing literature for neonates <3 Kg (8). Statistical analysis was conducted using SAS version 9.4 (SAS Institute, Cary, North Carolina), with a significance level set at *P* < 0.05.

## Results

**Figure 1:**
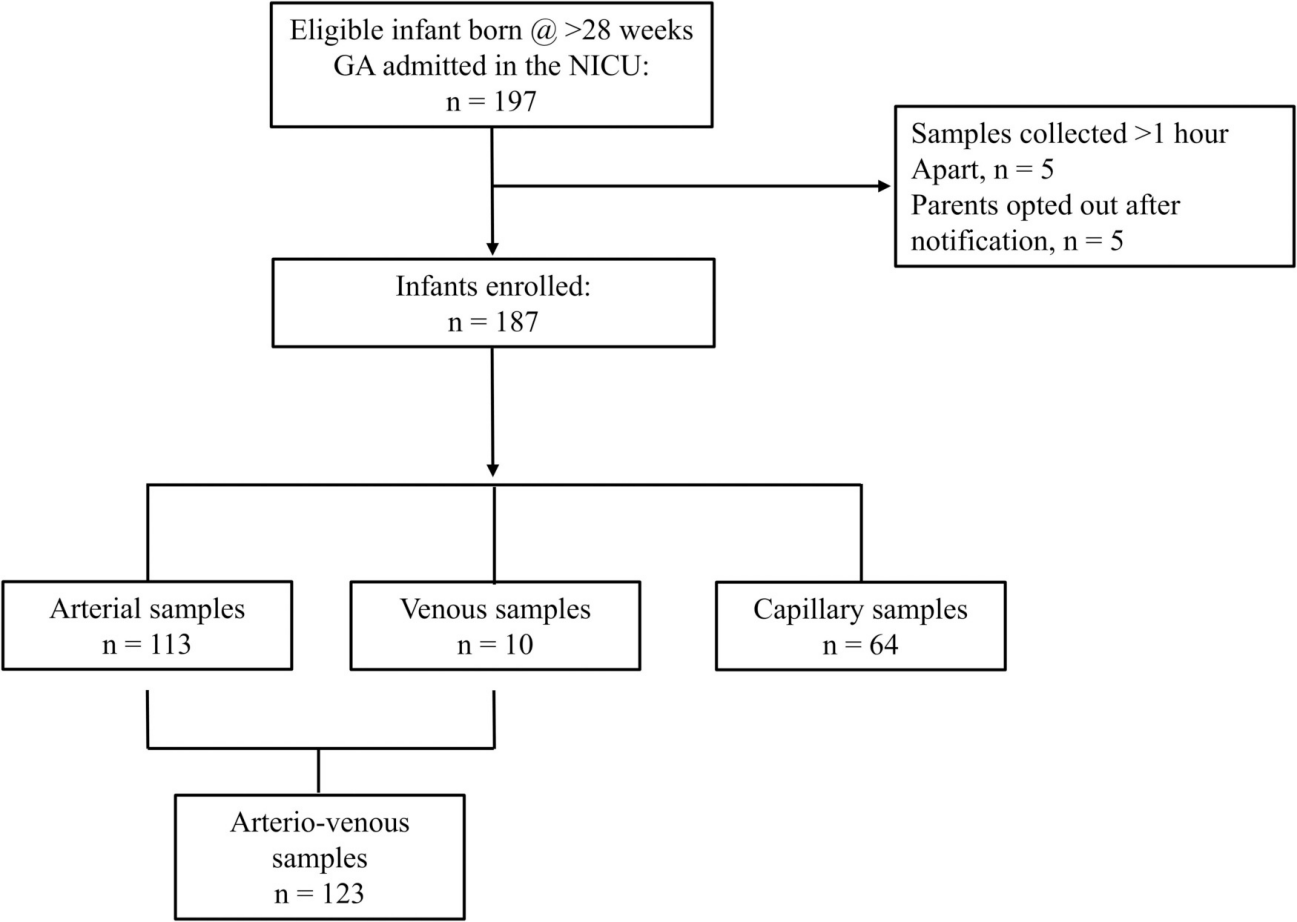
Flow diagram of study subjects.

During the study period, a total of 197 neonates were evaluated for eligibility, and 187 were successfully enrolled (**see** Figure 1). The demographic data of the cohort is detailed in Table 1. After the evaluation of the baseline characteristics, most of the infants were delivered through cesarean section 62.6% (117).

**Table 1:** Demographic characteristics of study infants (*n* = 187).

Independent Factors	Level	Number	Percentage
Gender	F	93	49.7
	M	94	50.3
Gestational age, wks. (Mean ± SD)	36.6 ± 3.2	187	100
Birth weight, gram (Mean ± SD)	2,835.5 ± 853.7	187	100
Race	Caucasian	122	65.3
	Black	12	6.4
	Hispanic	18	9.6
	Asian	11	5.9
	Other	24	12.8
Mode delivery	Vaginal	70	37.4
	C-section	117	62.6

F, Female; M, Male; C-section, Cesarean section; SD, Standard deviation.

### Comparing hemoglobin levels: central laboratory and POC testing measurements

In the capillary samples, the hemoglobin levels measured by POC testing were significantly higher than those obtained from laboratory testing. The POC testing showed values of 18.06 ± 2.07, while laboratory testing reported values of 17.65 ± 1.96, resulting in a 95% confidence interval of 0.09–0.72 and a mean difference of 0.41 (*p* = 0.01). In contrast, the arterio-venous samples revealed that hemoglobin levels measured by POC testing were slightly lower than those obtained from laboratory results, although this difference was not statistically significant. The POC testing recorded values of 16.04 ± 2.20, compared to 16.14 ± 2.29 in laboratory tests, with a 95% confidence interval of −0.29–0.1 and a mean difference of 0.09 (*p* = 0.34), as indicated in Table 2.

**Table 2:** Comparison of hemoglobin levels based on method of evaluation.

Variable	Min (g/dL)	Max (g/dL)	Mean	SD	Mean Diff	95% CI	*p*-value
Capillary Samples (*n* = 64)							
POC Hemoglobin	12.50	22.00	18.06	2.07	0.41	0.09–0.72	0.01
Laboratory Hemoglobin	12.30	21.30	17.65	1.96			
Arterio-venous samples (*n* = 123)							
POC Hemoglobin	6.80	23.00	16.04	2.20	0.09	−0.29–0.10	0.34
Laboratory Hemoglobin	6.80	22.80	16.14	2.29			

**Figure 2:**
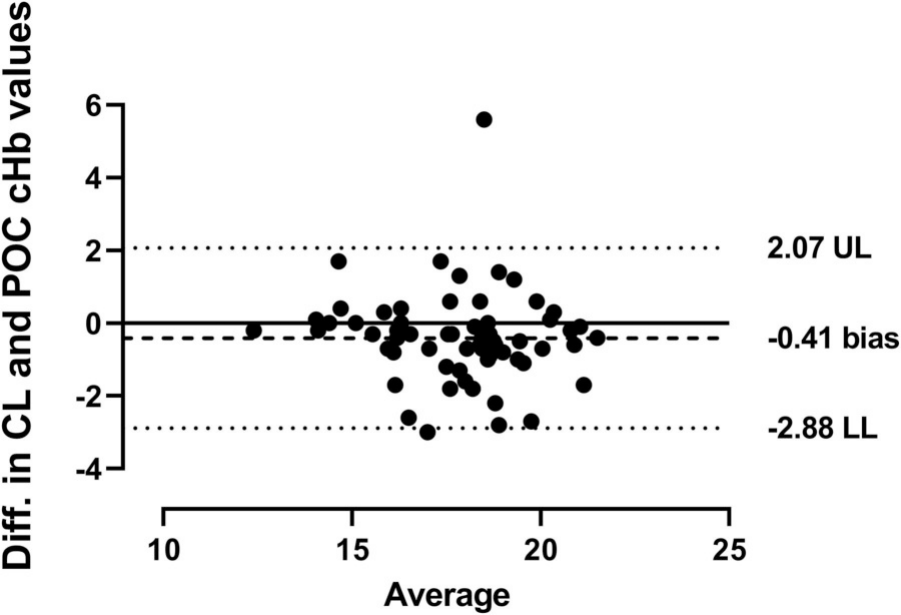
Comparison of central lab and POC capillary hemoglobin. The Bland-Altman plot shows agreement between central lab (CL) and POC capillary (c, capillary) hemoglobin values using linear regression and a 95% confidence interval. The solid line represents a perfect agreement, while the dotted lines indicate the limits of agreement at ±1.96 SD. UL, upper limit; LL, lower limit.

**Figure 3:**
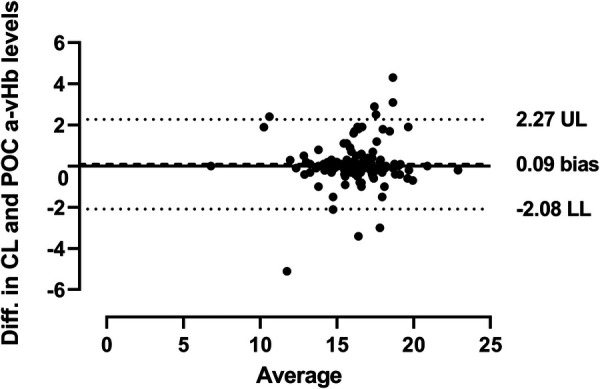
Comparison of central lab and POC arterio-venous hemoglobin. The Bland-Altman plot shows agreement between central lab (CL) and POC arterial-venous (a-v, arterio-venous) hemoglobin values using linear regression and a 95% confidence interval. The solid line represents a perfect agreement, while the dotted lines indicate the limits of agreement at ±1.96 SD. UL, upper limit; LL, lower limit.

The Bland-Altman analysis highlights the mean biases between POC testing and laboratory hemoglobin measurements. For capillary samples, the bias was −0.41, while for arterio-venous samples, it was 0.09. The lower limit of agreement (LL) for POC testing compared to laboratory hemoglobin was more negative for capillary samples (−2.88) than for arterio-venous samples (−2.08). Conversely, the upper limit of agreement (UL) was less positive for capillary samples (2.07) compared to arterio-venous samples (2.27), as shown in Figures 2, 3.

### Comparison of hemoglobin result turnaround times: point-of-care testing vs. central laboratory measurements

POC testing significantly reduces the time needed to obtain hemoglobin results, with an average of just 3.98 ± 7.1 min compared to 62.03 ± 41.75 min for central laboratory testing. This results in an average time difference of 58.05 ± 43.13 min (*p* < 0.0001; 95% CI, −64.27 to −51.83).

## Discussion

In clinical settings, hemoglobin values from both arterial and venous samples are commonly used laboratory parameters to monitor anemia and guide decisions regarding blood transfusions. In our prospective study involving 187 neonates born at a gestational age of over 28 weeks, we found that the results of POC testing for hemoglobin were comparable to those obtained from laboratory analyses of arterio-venous blood samples. However, we observed a significant discrepancy between capillary hemoglobin values measured by POC testing and those obtained through laboratory methods. These findings suggest that while POC testing may be a viable alternative for evaluating hemoglobin levels in arterio-venous samples, it may not accurately reflect hemoglobin values in capillary samples.

To our knowledge, this study is the first to prospectively compare POC testing with laboratory hemoglobin concentrations derived from the same or similar sources within an hour after birth for newborns born at a gestational age >28 weeks admitted to the NICU. Previous studies have reported variable results depending on the type of sampling, technique used, and population considered (8–10). Our observations with arterio-venous samples indicate that POC testing for hemoglobin levels is slightly lower compared to laboratory hemoglobin levels; however, there is good agreement with no significant difference when samples are taken from arterio-venous blood (mean difference of 0.09, *p* = 0.34). While Calandrino et al. found no similarity between arterio-venous POC testing and laboratory hemoglobin results, our findings align with those reported by Leino et al. and Zhang et al., suggesting that POC testing hemoglobin values from arterio-venous samples are slightly lower than laboratory values but with no significant difference (12, 15, 16).

Additionally, Bland-Altman analysis revealed that the mean difference between capillary POC testing, and laboratory hemoglobin levels displayed a statistically significant bias of −0.41 g/dL (*p* = 0.01), indicating that, on average, POC testing reported hemoglobin values were higher than those from laboratory testing at each comparison point in our samples (Figure 2). Because the ABL 90 Flex Plus lacks algorithmic adjustment or recalculation features to convert capillary values to estimated venous values, this difference in capillary specimens between POC and laboratory testing is unlikely to be related to matrix difference, but rather may be attributed to variability in mixing techniques, consistent with the study by Neville RG, which demonstrated a poor correlation between manually mixed samples by nurses and those analyzed by an automatic laboratory instrument in contrast to uniform mixing by laboratory staff using a mechanical mixer (17). Although the difference was statistically significant, it was less than our clinically significant difference of ±1 g/dL. Nevertheless, this discrepancy suggests caution should be exercised when using POC testing for capillary hemoglobin levels at very low or high hemoglobin levels. Thus, in neonates born at >28 weeks gestation, it is critical to obtain confirmatory laboratory hemoglobin testing when POC results are near these extremes.

Delays in obtaining hemoglobin results from laboratory tests can adversely affect patient care. At our institution, the turnaround time for laboratory hemoglobin results is approximately one hour. Notably, specimen transportation comprises 27.3% of this time, including 95 s via pneumatic tube system and 15 min from specimen collection to arrival at the hematology laboratory. This is where POC hemoglobin testing can significantly improve treatment strategies. In our study, the turnaround time for POC test results was substantially shorter than that of laboratory results, with a mean of 3.9 min compared to 62 min. This reflects an average difference of 58.05 ± 43.13 min (*p* < 0.0001). These findings are consistent with those of McCoy et al. (18), which indicated that POC testing yielded results in an average of 3.5 min, while laboratory results frequently took over 60 min. These results suggest that POC testing for hemoglobin can provide the rapid results needed to initiate early treatment, especially when using arterio-venous samples.

Several limitations of this study should be acknowledged. First, as a single-institution study, it may introduce biases related to specific practices and protocols. Conducting multicenter studies using ABL90 FLEX Plus POC instrument (Radiometer Medical ApS, Brønshøj, Denmark) could help validate the findings across different clinical settings. Second, although the study included 187 neonates, the population may not fully represent the broader neonatal population, especially in terms of racial diversity. Future studies with larger and more diverse samples could yield more generalizable results. Finally, we did not document the time taken to transfer the arterio-venous samples from the syringe to the specimen tube. While the transfers from syringes to specimen tubes were performed quickly, knowing the exact time for each transfer could provide additional clinical value, particularly in cases where the POC testing and laboratory hemoglobin levels differ. Therefore, a prospective multicenter study is needed to assess the impact of the timing of specimen transfer from the syringe to the specimen tubes in neonates.

The strengths of this study include comparing laboratory and POC testing methods for samples from the same or similar sources, which provide valuable insights into their relative effectiveness in a clinical setting. Additionally, examining the turnaround times of hemoglobin testing in neonates has significant implications for clinical practices in the NICU. Lastly, the study's prospective design allows for more reliable observations and conclusions.

## Conclusion

In conclusion, our study demonstrates that POC testing for hemoglobin levels in arterio-venous samples in neonates born at >28 weeks gestational age can provide results comparable to those obtained through laboratory analysis. This suggests that POC testing may be a viable option for rapidly evaluating hemoglobin levels, which is particularly beneficial for making timely clinical decisions. However, caution should be exercised when interpreting POC results from the capillary source. Thus, confirmatory laboratory testing is essential for capillary hemoglobin assessments given the potential clinical implications.

Future multicenter studies are warranted to validate these findings and further investigate the impact of varying measurement techniques and practices on the accuracy of POC hemoglobin testing across diverse settings.

## Data Availability

The original contributions presented in the study are included in the article/Supplementary Material, further inquiries can be directed to the corresponding author.
